# Supplementary figures and images for: The combined use of scRNA-seq and network propagation highlights key features of pan-cancer Tumor-Infiltrating T cells
Source: PLoS One. 2024 Dec 27;19(12):e0315980. doi: 10.1371/journal.pone.0315980 (PMC11676858; doi:10.1371/journal.pone.0315980)

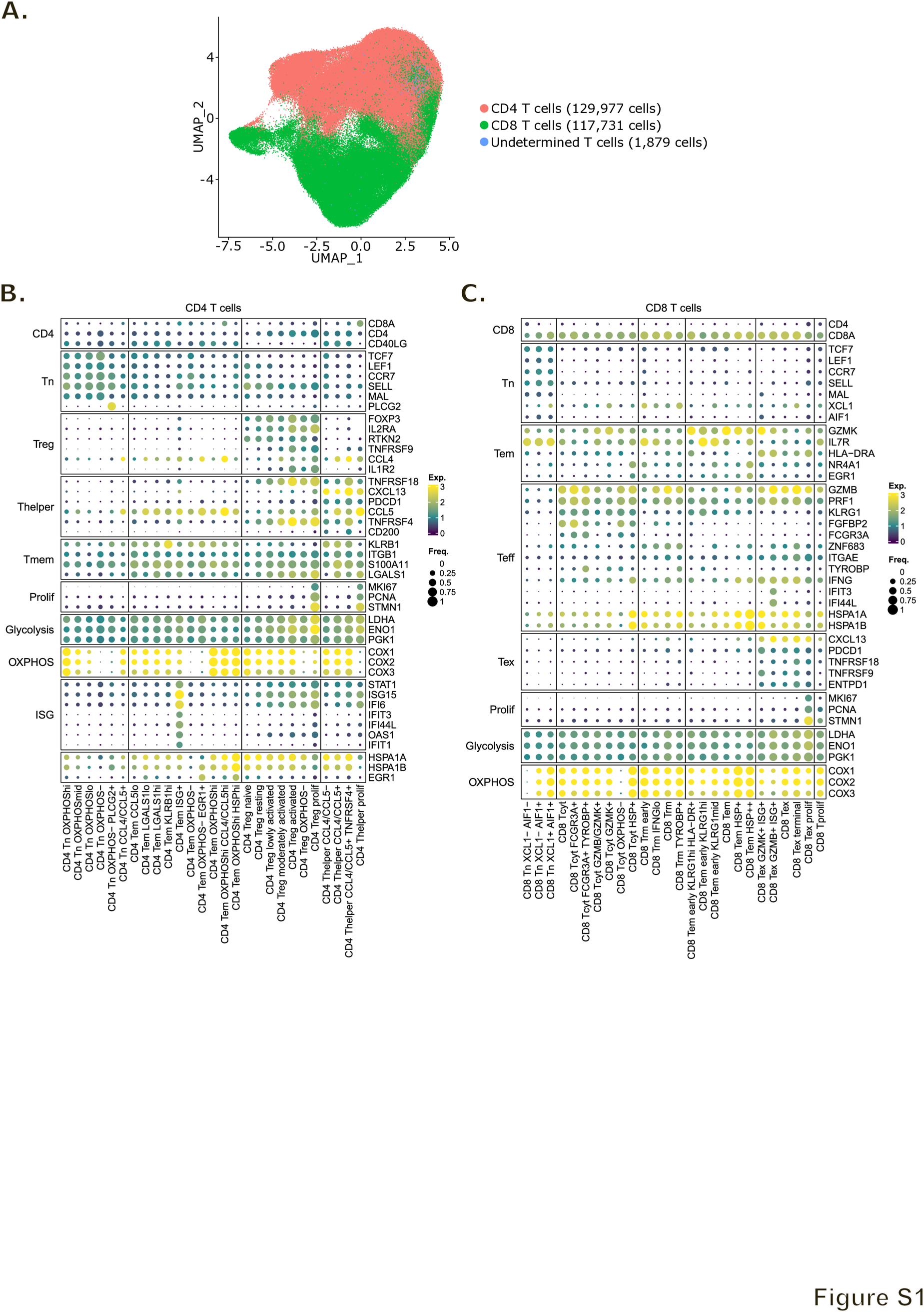

Supplement: S1 Fig — (A) UMAP representation of the T cell atlas colored by CD4+ or CD8+ subtype. (B, C) Bubble plot showing expression of representative markers genes of CD4+ (B) and CD8+ (C) T cell phenotypes. Color represents the normalized expression level and size represents the expression frequency. Prolif: proliferation, OXPHOS: oxidative phosphorylation, ISG: interferon-stimulated genes. (TIF) [file pone.0315980.s001.tif]

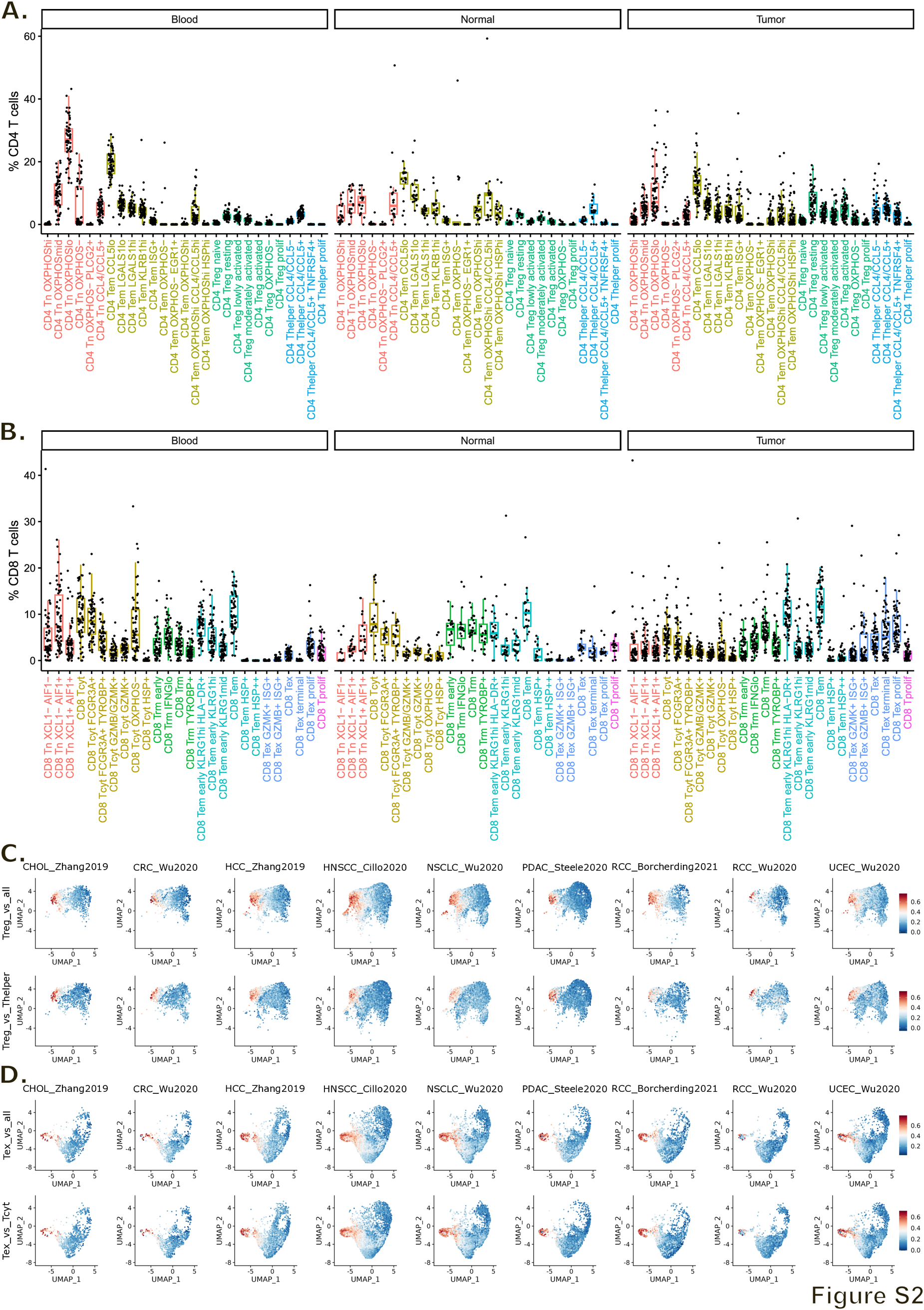

Supplement: S2 Ftsig — (A, B) Boxplot of CD4+ (A) and CD8+ (B) T cells detailed phenotype proportions compared to total CD4+ or CD8+ T cells in blood, adjacent normal tissue and tumor tissue. (C) UMAP representation of single cell Treg gene signatures scores in intra-tumor CD4+ T cells of the atlas. (D) UMAP representation of single cell Tex gene signatures scores in intra-tumor CD8+ T cells of the atlas. For (C) and (D), gene signature scores were computed using the AddModuleScore function from Seurat package with default parameters. (TIF) [file pone.0315980.s002.tif]

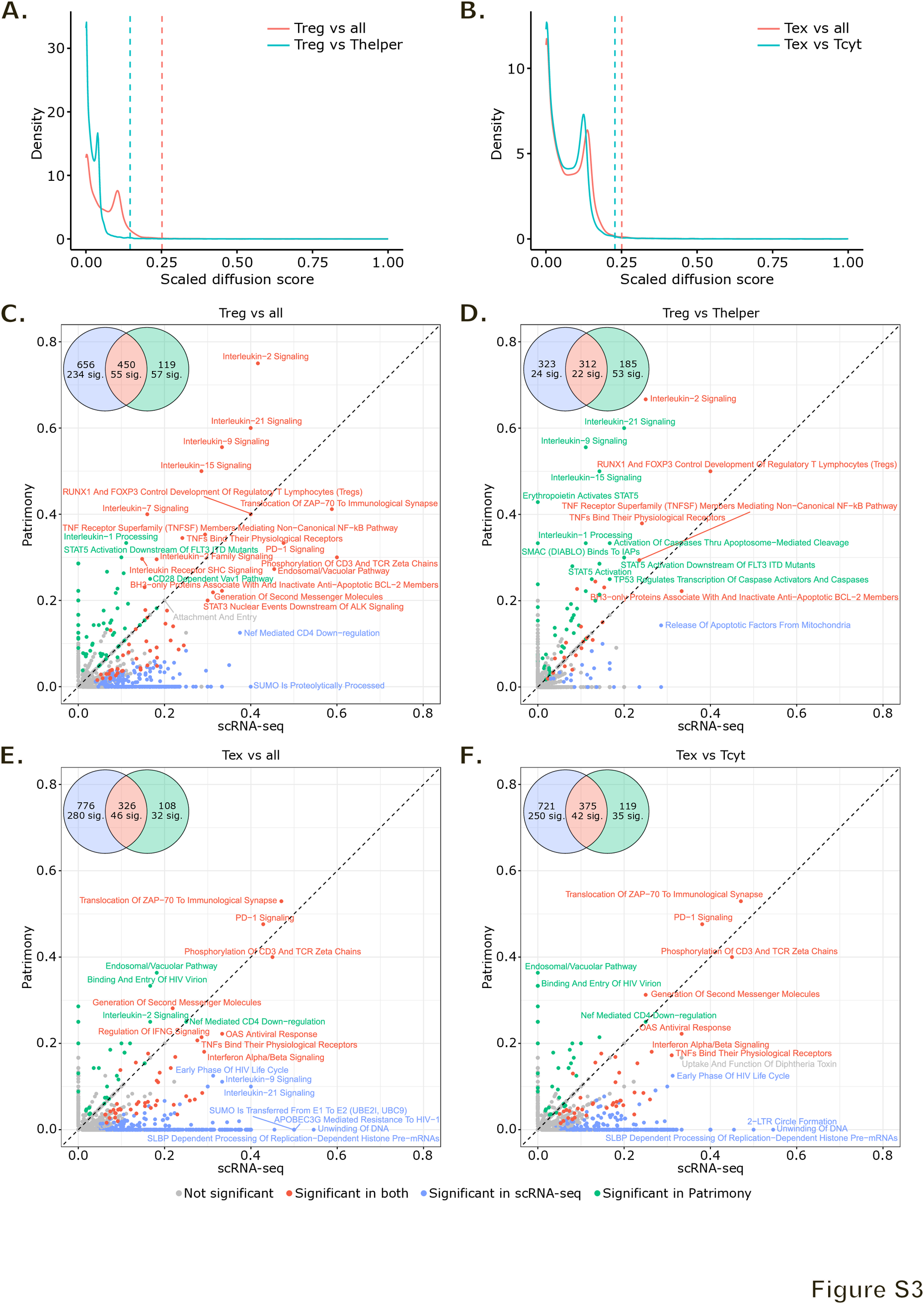

Supplement: S3 Fig — (A, B) Density plot of scaled diffusion scores computed with Patrimony for TI-Treg (A) and Tex (B) signatures. Vertical dashed lines correspond to the top 200 gene value for indicated signatures. (C-F) Concordance plots and Venn diagrams comparing Patrimony and scRNA-seq signatures for “Treg vs all” (C), “Treg vs Thelper” (D), “Tex vs all” (E) and “Tex vs Tcyt” (F). Venn diagrams show represented and significantly (sig.) represented pathways. Pathways with adjusted p-value < 0.05 were considered significant. Concordance plots compare gene ratios for each represented pathway. The black dashed line represents the first bisector (y = x). (TIF) [file pone.0315980.s003.tif]
